# An Investigation into the Properties and Microstructure of Cement Mixtures Modified with Cellulose Nanocrystal

**DOI:** 10.3390/ma10050498

**Published:** 2017-05-04

**Authors:** Jessica Flores, Mahsa Kamali, Ali Ghahremaninezhad

**Affiliations:** Department of Civil, Architectural and Environmental Engineering, University of Miami, Coral Gables, FL 33146, USA; j.flores7@umiami.edu (J.F.); m.kamali@umiami.edu (M.K.)

**Keywords:** CNC, cement mixture, hydration, microstructure

## Abstract

This paper aims to examine the effect of cellulose nanocrystals (CNC) on the hydration, transport behavior, and microstructure of cement mixtures. The addition of CNC delayed hydration at an early age but improved hydration at later ages. A small increase in the electrical resistivity of the cement mixtures with CNC was observed. Statistical nanoindentation showed a small tendency to a larger volume fraction of high density calcium-silicate-hydrate (C-S-H) and a smaller volume fraction of low-density C-S-H in the mixture with CNC.

## 1. Introduction

Cellulose nanocrystal (CNC) is receiving increasing attention as nanofiber-reinforcements in composites in a wide array of applications [1,2,3,4,5,6,7,8]. This is due to its superior physical properties (including low density and high aspect ratio), chemical characteristics (such as being surface functionalizable), and high axial elastic modulus [9,10]. CNC is derived from the chemical treatment of cellulose fibers, which comprise the structure of trees and plants [9,10]. Thus, the intrinsic sustainable nature and the nanosize characteristic of CNC have the potential to benefit cement-based materials to improve the strength and durability and reduce carbon footprint of civil engineering materials. Prior investigations examined the effect of nanomaterials such as nanosilica [11,12,13,14,15,16], titanium dioxide nanoparticles [17,18,19,20,21,22,23,24,25,26,27,28], carbon nanotubes [29,30,31] and nanoclay [32,33,34,35] on the performance of cement-based materials. In spite of intense research into the use of CNC in the fabrication of nanocomposites in other disciplines [1,2,3,4,5,6], the use of CNC in cement-based materials has not been investigated until very recently in three publications by Cao et al. [36,37,38]. In their work, the authors showed improvements in the hydration, flexural strength, and microstructure of cement mixtures [37,38]. Using nanoindentation, they indicated an increase in the stiffness of high density C-S-H surrounding unhydrated cement particles [38].

In spite of possible benefits resulting from the use of CNC in cement-based materials, related work on this topic seems to be very scarce and limited to the three recent publications mentioned above. In addition, there is variation in the size distribution and surface chemistry of CNC arising from different processing routes and chemical modifications. Thus, this study aims to investigate the effect of CNC with a size distribution different from that used in the studies by Cao et al. The effect of CNC on the hydration was studied using chemical shrinkage test and semi-adiabatic calorimetry at early age and non-evaporable water content measurement at later ages. The chemical analysis of the hydration product was performed with the aid of X-ray diffraction (XRD) and Fourier transform infrared spectroscopy (FTIR). The electrochemical impedance spectroscopy (EIS) method was used to evaluate electrical resistivity as an indication of the transport behavior of the modified mixtures. Microstructural examination was performed with scanning electron microscopy (SEM). Nanoindentation was conducted to study the change in the nanomechanical properties of C-S-H as a result of CNC addition. The nanoindentation technique has been successfully utilized in the past for the characterization of the nanomechanical properties and microstructure of cementitious materials [30,31,39,40,41,42,43].

## 2. Experiments

### 2.1. Materials and Specimen Preparation

**Figure 1:**
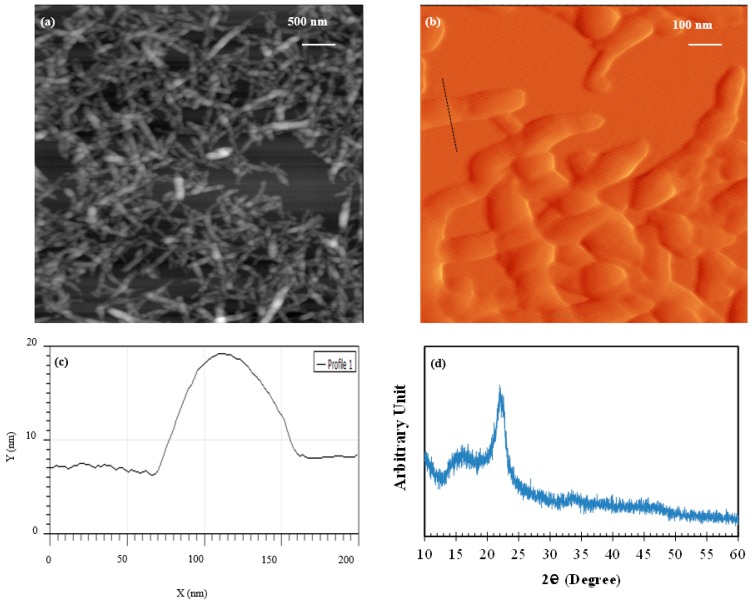
(**a**,**b**) Height and error atomic force microscopy (AFM) images, respectively, of cellulose nanocrystals (CNC) taken at different magnifications; (**c**) Line profile of a CNC nanofiber along the line marked with a dashed line in Figure 1b; (**d**) XRD spectrum of CNC.

In this paper, CNC was purchased in the form of a 7.4% by mass aqueous suspension (BGB Natural, Blue Goose Biorefineries Inc., Saskatoon, SK, Canada). Per the manufacturer’s specification, the particle size measurement of CNC using dynamic light scattering showed a bimodal distribution with nominal average sizes of about 90 nm and 700 nm corresponding to the width and length of CNC, respectively. The surface charge of CNC was −33.4 mV. In order to obtain insight into the morphology of CNC, atomic force microscopy (AFM) was used to image CNC in this study. To this end, a small amount of CNC was added to deionized (DI) water, stirred vigorously for 30 min, and then ultrasonicated for an hour. A drop of the dispersed CNC was cast on a freshly cleaved mica surface and allowed to dry completely. The dried mica surface was scanned with an atomic force microscope (TT-AFM from AFMWorkshop, Signal Hill, CA, USA) using a silicon probe (ACLA from AppNano, Mountain View, CA, USA) with a spring constant of 36–90 N/m and tip radius of less than 10 nm based on the specifications provided by the manufacturer. An AFM height image and error image at different magnifications are shown in Figure 1a,b, respectively.

A line profile of a CNC nanofiber is depicted in Figure 1c. The line is marked with a dashed line in Figure 1b. It is seen that the CNC appear to have a width of about 90–120 nm and a variable length of about 500 nm. Since light scattering uses a larger volume than can be imaged in AFM, it provides a more realistic size representation of CNC. Nonetheless, the size estimates from AFM and light scattering are qualitatively in agreement. Although not explicitly indicated, the size of CNC used in [36,37,38] was implied to have a width of about 3–5 nm and length of about 200 nm. The XRD spectrum of CNC is illustrated in Figure 1d. The diffraction peaks seen at 2*θ* of 16.8° and 22.6° are associated with native cellulose [44].

Cement mixtures were formulated using a type I/II Portland cement with addition of CNC at rates of 0.4% and 0.8%, per mass of cement. Table 1 shows the oxide composition of the cement. The water/cement ratio of the mixtures was 0.4. CNC was first ultrasonicated in water for about 30 min to achieve good dispersion. Then, the dispersed CNC was added to a bucket containing water and cement and the mixture was mixed in the bucket for about 30 s with a mixer at a slow rate. Then the mixing was stopped and the inside surface of the bucket was scraped for about 15 s. Then, mixing was continued for another 60 s at a medium speed.

Specimens in the shape of cubes with a dimension of 50 mm were cast in steel molds. One layer of the mixture was placed to occupy half the space of the mold and then tamped 32 times. Then the mold was fully filled with a second layer of the mixture and tamped again to aid consolidation. The molds were covered using a plastic sheet and placed in a curing room with a relative humidity of more than 95% and temperature of about 23 ± 2 °C. Cubes were removed from the molds after 24 h and cured in a saturated solution of calcium hydroxide.

### 2.2. Chemical Shrinkage

The reduction in volume between the hydration product and the initial reactants in the hydration reaction is referred to as chemical shrinkage. Thus, chemical shrinkage measurements can be related to the hydration rate at early age in cement-based materials. Mixtures with and without additions of CNC were formulated with a water/cement ratio of 0.4 in accordance with ASTM C 1608. The mixtures were inserted into a slender glass vial to occupy about 5–10 mm of the glass vial. De-aerated water was added on top of the cement mixture to occupy the remaining portion of the vial. Rubber stoppers were used to seal the vials. A capillary tube was passed through the stopper and a drop of oil was placed on top of the water column in the capillary tube to avoid water loss due to evaporation. The height of the water column was monitored for one day and the chemical shrinkage (mL/g cement) was determined from the following formula:

(1) $$\text{Chemical Shrinkage}\;=\;\frac{\left[H-H_{1}\right]}{M}$$

where H and $H_{1}$ are the height of the water column at different times and at one hour, respectively, and *M* is the cement mass (g) used in the glass vial. Duplicate samples were used for each mix design and the average value was determined.

### 2.3. Heat of Hydration

The hydration progress of the mixtures at early age was monitored using the semi-adiabatic calorimetry technique. Hydration temperature measurement was conducted utilizing a semi-adiabatic calorimeter (AdiaCal, W.R. Grace, Cambridge, MA, USA) at a sampling rate of 1/min. About 300 g of fresh mixtures with a water/cement ratio of 0.4 were prepared per ASTM C 305 for the measurement. The time between initial sample mixing and placement in the instrument was kept to less than five minutes.

### 2.4. Non-Evaporable Water Content (NEWC)

The portion of water that is chemically bound to the hydration product is an indicator of the hydration progress. The *NEWC* of the hydration product obtained from loss on ignition is used to evaluate the degree of hydration. For this test, samples in the form of pulverized cement mixture finer than the sieve # 60 were prepared. Pulverized samples with a mass of about 5 g were placed for one day in an oven set at 105 °C to remove all the evaporable water. Then samples were transferred into a muffle furnace set at a temperature of about 1000 °C. The ignition at this temperature for three hours removes chemically bound water from the pulverized sample. The non-evaporable water content per cement mixture mass, NEWC (%, g/g cement mixture), was calculated from the following equation:

(2) $$NEWC=(\frac{M^{I}-M^{II}}{M^{II}}-LOI)\times 100$$

where $M^{I}$ and $M^{II}$ are the mass of the pulverized samples after removing evaporable water and after removing chemically bound water, respectively, and *LOI* is the cement’s loss on ignition.

### 2.5. X-ray Diffraction (XRD)

In this study, XRD was used to examine the influence of CNC addition on the chemical composition of the hydration product. Pulverized samples finer than the sieve # 60 were prepared for this test. Moisture was removed from the samples by storing them for one day in a vacuum oven set at a temperature of 105 °C. Use of vacuum oven prevented samples from carbonation. The XRD instrument consisted of a Siemens 5000D X-ray diffractometer using a radiation source of Cu K*α*. The parameters used for scanning were a step size of 0.02 degrees and scan rate of one degree per minute.

### 2.6. Fourier Transform Infrared Spectroscopy (FTIR)

The FTIR analysis was utilized to study the composition of the hydrated cement and possible interaction between CNC and the hydrated cement. For FTIR measurement, pulverized samples obtained from the control mixture and the mixture with 0.8% CNC at the age of 28 days were prepared in a similar way to the XRD measurement. Another set of samples consisting of a suspension of 1 g of cement and 0.04 g of CNC in 200 g of distilled water was prepared. The use of a high mass (relative to cement mass) of CNC in the suspension compared to that in the cement mixture was meant to increase the possibility of interaction between CNC and hydration product and allowed one to investigate such interaction with FTIR. A control suspension without CNC addition was also prepared. The suspensions were filtered after 3 h and dried in the same way as the pulverized samples from mixtures. All samples were scanned in a PerkinElmer Paragon 1000 instrument in the range of 650 cm^−1^ to 4000 cm^−1^ with a 0.1 cm^−1^ resolution.

### 2.7. EIS Measurement

In this study, EIS was utilized to evaluate the electrical resistivity of the mixtures. Due to strong dependence of electrical resistivity on the microstructural features and transport characteristics, use of electrical resistivity measurement as an indicator of durability performance of cement-based materials has gained increasing attention [45,46,47,48,49,50,51,52,53,54]. Cube samples were placed between two conductive electrodes and their electrical resistivity was measured using a Gamry Reference 600. The EIS measurement parameters used in this test were a 250 mV alternating signal and the 10E6-10 hertz frequency domain. The average electrical resistivity of three samples per each mix design was determined using the dimensions of the cubes.

### 2.8. Microscopic Examination

In order to gain insight into the effect of CNC addition on microstructure, the control mixture and mixture with 0.8% CNC at 28 days of curing were examined in a scanning electron microscope. Samples for SEM examination were prepared by embedding small pieces of oven dried, hardened mixtures in an epoxy and then polishing the surface using abrasive papers with decreasing roughness starting from 180 grit size to 1200 grit size. Ethanol was used as the polishing fluid and also used for cleaning the surface of the samples after polishing with each abrasive paper. Polishing was continued using a one micron diamond paste followed by cleaning with ultrasonication in ethanol for about 15 min. SEM samples were then coated with a conductive layer and examined in the backscatter mode.

### 2.9. Nanoindentation Measurement

Nanoindentation was utilized to evaluate the effect of CNC addition on the mechanical response of the hydration product at the nanoscale. Since C-S-H is the primary binding phase in the hydration product, understanding the influence of CNC on the nanomechanical behavior of C-S-H is important. In this study, nanoindentation was performed using an AFM. In an AFM indentation test, a cantilever probe with a known stiffness comes into contact with the surface of the material, indents to a certain depth, and then retracts from the surface. The deflection of the beam versus vertical position of the cantilever tip is obtained using a sensitivity factor. The sensitivity factor is calculated as the slope of a linear fit to the voltage-distance curve when the same probe is pushed against a relatively hard surface compared to the tested material, so that the indentation depth can be assumed to be negligible. Deflection is converted to force having the spring constant of the cantilever using Hooke’s law to obtain the force-indentation distance curves. The force-indentation distance curve corresponding to the unloading was fitted to the Hertzian contact model [55] as follows:

(3) $$F=\frac{4}{3}MR^{0.5}\delta^{1.5}$$

(4) $$\frac{1}{M}=\frac{1-\nu_{1}^{2}}{E_{1}}+\frac{1-\nu_{2}^{2}}{E_{2}}$$

where *F* is force, *R* is the tip radius, *δ* is indentation distance, *M* is indentation modulus, $\nu_{1}$ and $\nu_{2}$ are the Poisson’s ratios, and $E_{1}$ and $E_{2}$ are the Young’s moduli of the sample and tip, respectively. The elastic constants of the tip material are known, and thus the above equation allows determination of the Young’s modulus of the sample. A cube corner diamond probe (DNISP from Bruker) with spring constant of 291 N/m and tip radius of 40 nm based on the specification provided by the manufacturer was used for AFM indentation. Indentation tests were performed on 16 random grids of 3 × 3 points on each sample. The spacing between points of each grid was 5 μm. The deflection sensitivity was calibrated using a sapphire substrate from Bruker.

## 3. Results and discussion

### 3.1. Chemical Shrinkage

**Figure 2:**
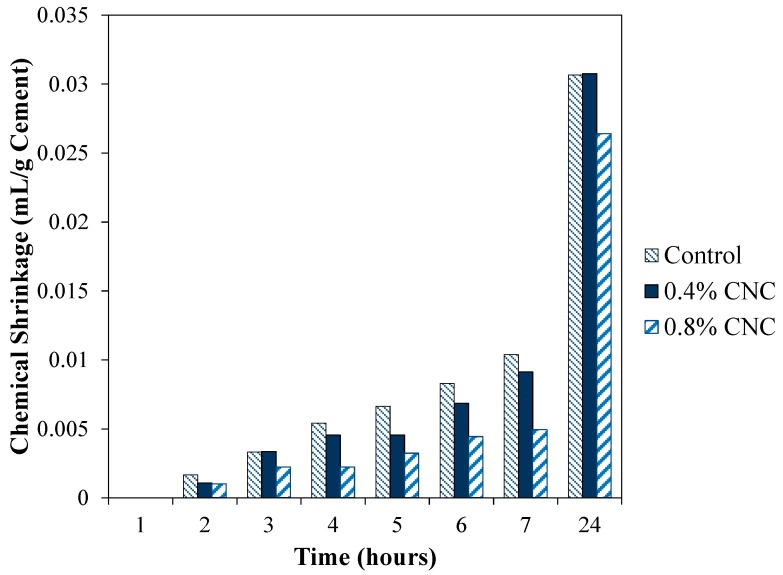
Chemical shrinkage of the mixtures.

The chemical shrinkage results of the mixtures, normalized per mass of cement, are shown in Figure 2. The product of the hydration reaction contains less volume than the initial reactants; thus, chemical shrinkage provides a measure of hydration rate in cement-based materials [18]. The control mixture showed a higher chemical shrinkage than the mixtures modified with CNC in the first seven hours. At 24 hours, the chemical shrinkage of the control mixture is higher than that of the mixture with 0.8% CNC.

Use of nanoparticles usually accelerates the hydration process of cement due to their large surface area increasing nucleation sites for the hydration product [18,56]. This effect is effective if the nanoparticles are uniformly dispersed within the space between the cement particles in the mixture. However, it is seen from the results shown in Figure 2 that an opposite effect is observed. A possible explanation for such behavior could be related to the interaction between the cement particles and CNC. In case the interaction forces between the cement particles and CNC favor the adsorption of CNC onto cement particles, a delay in the hydration of cement particles is expected. This is due to a reduction in available surface area of cement particles to participate in the hydration reaction. In order to investigate the potential adsorption of CNC onto the cement particles, the surface charge of CNC and cement particles were compared. The surface charge of the CNC used in the experiments was about −33.4 mV obtained using zeta potential measurement per the manufacturers’ specifications. The surface charge of cement particles from available literature is about −10 mV [57,58]. Due to a larger surface charge of CNC compared to cement particles, the likelihood of agglomeration of CNC particles is less than their adsorption onto cement particles. A similar mechanism was suggested by [37] regarding the effect of CNC on early age hydration of cement particles. Thus, it is likely that adsorption of CNC onto cement particles is responsible for a reduction in the chemical shrinkage of the cement mixture modified with CNC.

### 3.2. Semi-Adiabatic Calorimetry

**Figure 3:**
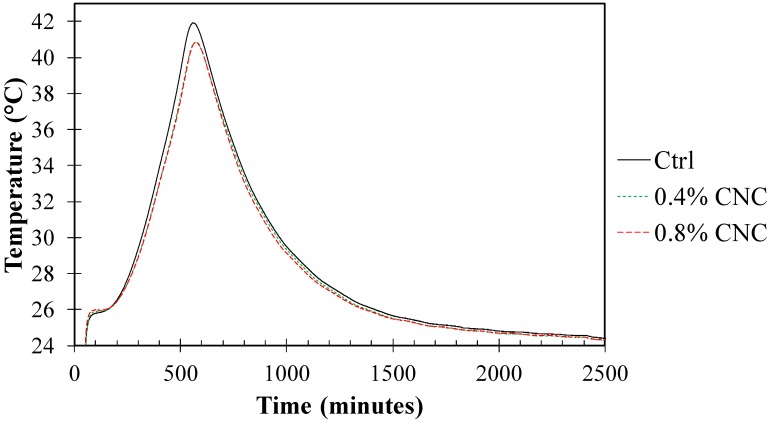
Hydration temperature curves of the mixtures obtained from semi-adiabatic calorimetry.

The hydration temperature curves of the control mixture and the mixture with 0.4% and 0.8% CNC are shown in Figure 3. It is seen that the control mixture had a slightly higher temperature peak compared to the mixture with 0.4% and 0.8% CNC. This suggests a tendency to a delay in early age hydration in the cement mixtures as a result of CNC addition. The difference in the early hydration between the cement mixtures modified with 0.4% CNC and 0.8% CNC, as observed in the results from the chemical shrinkage test, was not observed in the hydration temperature curves. This may be attributed to the difference in the nature of the measurement technique for early hydration evaluation.

### 3.3. Non-Evaporable Water Content (NEWC)

**Figure 4:**
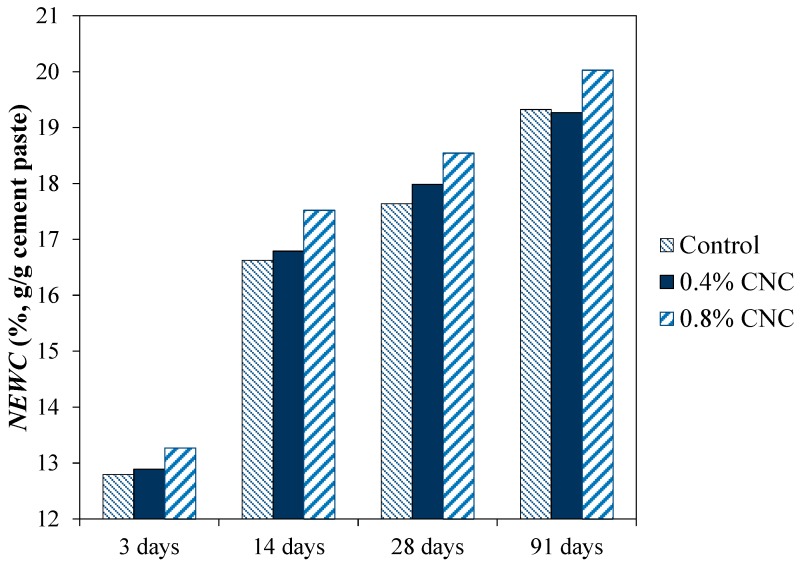
Non-evaporable water content (NEWC) of the mixtures at various ages of curing.

The results of *NEWC* measurement, normalized by the mixture mass, of mixtures at varied ages of curing are presented in Figure 4. It is seen that the mixture with 0.8% CNC showed a slightly higher *NEWC* compared to the control mixture. The control mixture and the mixture with 0.4% CNC showed a similar NEWC. It is interesting to note the increase in hydration after an initial reduction as observed from the semi-adiabatic calorimetry and chemical shrinkage results.

The enhanced hydration could be due to the influence of CNC adsorption onto cement particles. The adsorption of CNC generates a steric repulsion between cement particles reducing agglomeration of cement particles, which in turn improves the hydration reaction. Additionally, it is possible that the seeding effect of CNC particles, which increases the nucleation sites for hydration reaction contributes to hydration improvement. As mentioned previously, the examination of the surface charge of CNC and cement particles suggests that the likelihood of CNC agglomeration is less than cement particle agglomeration or cement particle/CNC agglomeration; therefore, it is likely that some of CNC particles are still dispersed in the microstructure and are able to impart their seeding effect. In this case, the dosage of CNC and the water/cement ratio of the mixture are expected to influence the dispersed amount of CNC in the mixture. This mechanism could explain the slightly higher degree of hydration in cement mixture with 0.8% CNC than the cement mixture with 0.4% CNC. It should be noted that the improvement in hydration as a result of CNC addition was more pronounced in [37]; this could be due to a smaller size of CNC or different surface characteristics of CNC used in their study. Thus, the results presented here highlight the importance of CNC size and surface chemistry on their influence on the properties of cementitious materials.

### 3.4. XRD Analysis

**Figure 5:**
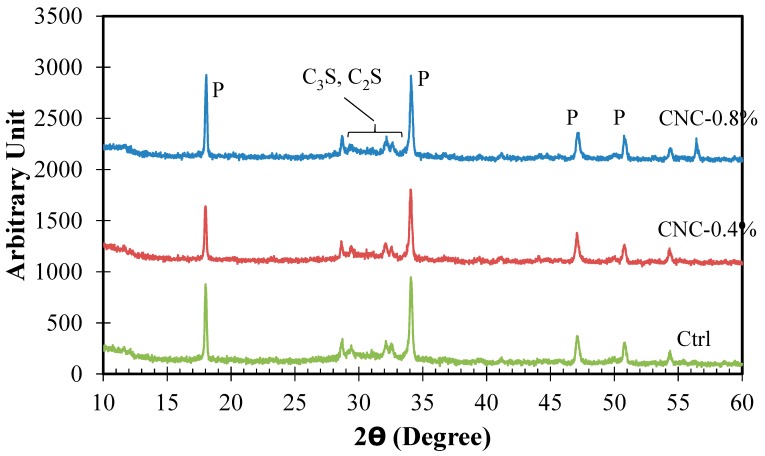
X-ray diffraction (XRD) spectra of the cement mixtures at 28 days of curing. P denotes portlandite.

The diffraction spectra corresponding to the control mixture and the mixtures with 0.4% and 0.8% CNC at 28 days are illustrated in Figure 5. The peaks at 2*θ* of 18.00°, 34.10°, 47.12°, and 50.81° are attributed to portlandite (P) [59]. The peaks in the 2*θ* range of 29°–33° are attributed to tricalcium silicate (C_3_S) and dicalcium silicate (C_2_S) [59], which comprise the main constituents of the unhydrated cement particle.

The XRD results are useful in the qualitative investigation of the phase composition of cementitious materials. Statistically significant changes in the peak values of crystalline phases, such as Ca(OH)_2_ and CaCO_3_, as well as appearance of new peaks are indicative of a change in the phase composition of existing phases or formation of new phases, respectively, in the microstructure. The comparison of the spectra of the cement mixtures does not show the presence of any new phases in the microstructure as a result of CNC addition within the resolution of the measurement. The P peaks at 18.00° and 34.10° of the cement mixture with 0.4% CNC appear to be slightly smaller than those of the control mixture. This difference is not evident in the spectrum of the cement mixture with 0.8% CNC. However, it should be noted that XRD measurements are qualitative and any definitive conclusions should be made with caution. Thus, it can be concluded from the XRD spectra that no significant difference can be observed in the phase composition of the cement mixtures modified with CNC compared to the control mixture.

### 3.5. FTIR

**Figure 6:**
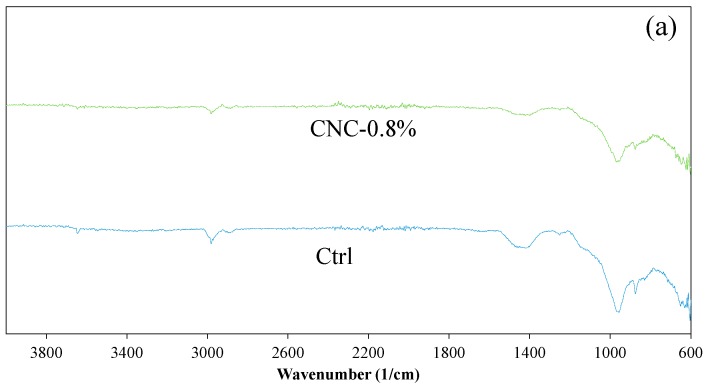

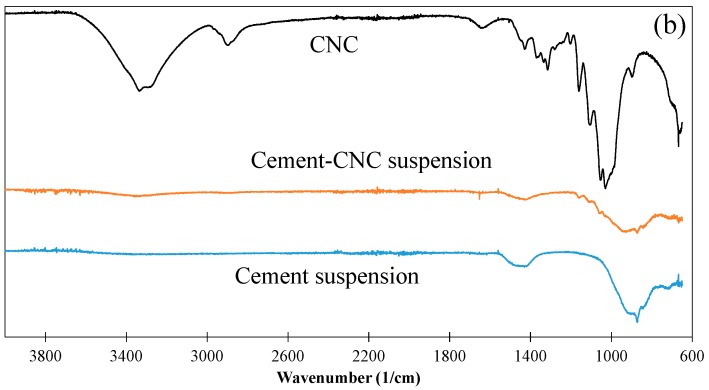
(**a**) Fourier transform infrared spectroscopy (FTIR) spectra of the control and the 0.8% CNC modified mixtures at 28 days of age; (**b**) FTIR spectra of pure CNC, cement suspension, and cement-CNC suspension.

The FTIR spectra of the control mixture and the mixture with 0.8% CNC at the age of 28 days are shown in Figure 6a. The peak at 3646 cm^−1^ appearing in both scans corresponds to hydroxyl group in Ca(OH)_2_ [29,60]. The band at 970 cm^−1^ is attributed to Si-O stretching in C-S-H [29,60,61]. The bands seen at 1410 and 870 cm^−1^ are associated with the presence of calcium carbonate [29,60]; although care was exercised to prevent contact of the samples with air, carbonation appeared to occur in the samples. However, since pronounced signature peaks of crystalline calcium carbonate are not present in the XRD spectra, it is likely that only small carbonation occurred. Similar to the XRD measurements, a comparison of the FTIR spectra of the control cement mixture and the cement mixtures with 0.8% CNC suggests a similar chemical composition of the microstructure in these two cement mixtures.

The FTIR spectra of pure CNC, cement suspension, and cement-CNC suspension are presented in Figure 6b. As previously mentioned, use of a cement-CNC suspension with an increased mass fraction (relative to cement mass) of CNC was meant to increase the potential interaction between hydration product and CNC. The broad band in the CNC spectrum in the range of 3100–3700 cm^−1^ is attributed to the hydroxyl group of CNC. The peak in the CNC spectrum at 670 cm^−1^ represents carboxylic acid group (COOH) bending [62]. The most notable observation here relates to the disappearance of hydroxyl group in the cement-CNC suspension spectrum, which could indicate formation of bonds between the hydroxyl groups of CNC and the hydration product. Such interactions could have potential implications in the load transfer between CNC and the hydration product affecting the mechanical behavior of the cement mixture.

### 3.6. Electrical Resistivity

**Figure 7:**
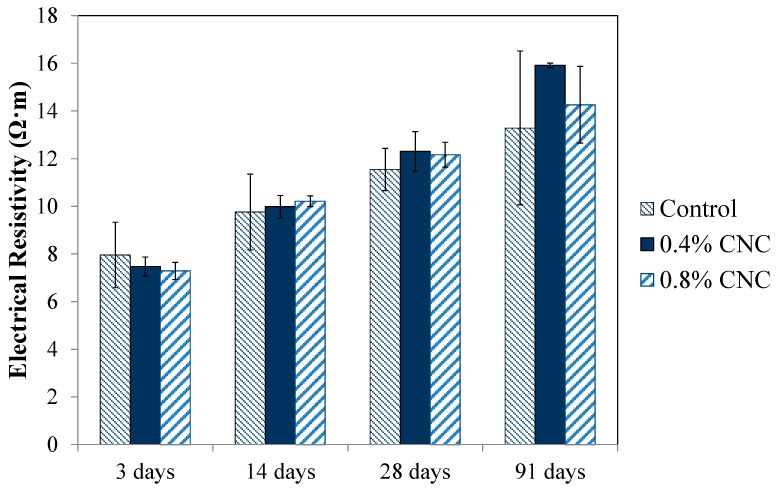
Electrical resistivity of the control mixture and the mixtures with 0.4% and 0.8% CNC at various ages of curing.

The results of the EIS measurement showing the electrical resistivity of the mixtures are presented in Figure 7. The electrical resistivity increased with age in all mixtures as the hydration continued and more hydration product occupied the pore spaces in the microstructure. It is seen that the electrical resistivity of the mixtures with CNC is slightly lower at three days, but it improves with age and exceeds that of the control mixture at late ages. However, the differences in the electrical resistivity considering the standard errors are small. The electrical resistivity of cementitious materials is governed by the pore structure and pore solution chemistry and ionic mobility [47,63]. Improved microstructure as a result of increased hydration, as shown from the non-evaporable water content measurements, could be suggested as a factor contributing to increased electrical resistivity of the mixtures with CNC compared to the control mixture. In addition, the presence of CNC with negative surface charge could increase the binding of cations in the pore solution, deceasing their mobility. As a consequence, an increase in the electrical resistivity is likely to occur. However, a detailed study is needed to elucidate the validity and the effect of this hypothesis.

### 3.7. Microscopic Examination

**Figure 8:**
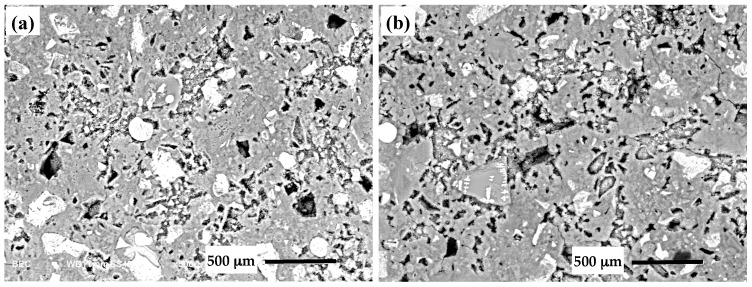
Scanning electron microscopy (SEM) images of the (**a**) control mixture and (**b**) the mixture with 0.8% CNC at 28 days of age.

The micrographs of the control mixture and the mixture with 0.8% CNC at 28 days of curing are demonstrated in Figure 8. Since the SEM images were taken in the backscatter mode, the contrast in the brightness of the images can be used to distinguish between phases with different atomic numbers. The unhydrated cement particles are viewed bright, the capillary pores appear dark, and the hydration products are seen gray in the images. Some microcracks (not shown here) were observed in the microstructure of both mixtures. It is possible for some microcracks to form due to sample preparation for SEM examination. An image analysis was performed to evaluate the microstructure of the two mixtures. To this end, SEM images in the backscatter mode were segmented in the software ImageJ and analyzed using the overflow method [64,65] to identify the pores and unhydrated cement particles. The porosity was determined as the total area fraction of pores in each image and the average of ten images was calculated. The porosity of the control mixture and the mixture modified with 0.8% CNC was determined to be 15.7 ± 0.6 and 16.9 ± 1.1, respectively, which indicates an insignificant difference in the total porosity of the two mixtures. The comparison of the area fraction of unhydrated cement particles indicated a small reduction in the area fraction of unhydrated cement particles in the mixture with CNC compared to the control cement mixture. Since the area fraction of unhydrated cement particles is related to the degree of hydration, the cement mixture with 0.8% CNC seems to show a slightly higher degree of hydration than the control mixture. This observation is in a general agreement with the results from the non-evaporable water content measurement shown in Figure 4.

### 3.8. Nanoindentation

The multiphase microstructure of the hydration product in cement mixtures consists primarily of C-S-H, Ca(OH)_2_, and a porous phase [30,66]. Thus, the results of the nanoindentation measurement at various locations in the microstructure exhibit a composite signature of various phases in the microstructure. Statistical analysis methods have been introduced to deduce mechanical properties and volume fraction of different phases in the microstructure [42,67].

**Figure 9:**
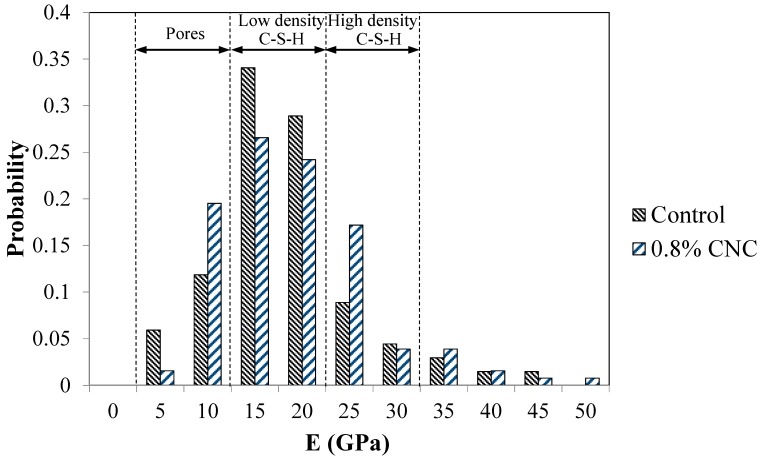
Nanoindentation results showing the probability distribution of the Young’s modulus of the mixtures corresponding to 28 days of curing.

The results of the statistical nanoindentation measurements on the control mixture and the mixture with 0.8% CNC at 28 days are shown in Figure 9. According to the available data in the literature, the values of the Young’s modulus larger than 50 GPa are considered to correspond to unhydrated cement particles [66,68] and are not shown in the figure. Values less than 10 GPa are typically associated with pores [30]. Depending on the packing density of C-S-H, two distinct phases, high density C-S-H and low density C-S-H, are considered [30,66,68]. The range of Young’s modulus corresponding to these two phases of C-S-H are delimited in the figure [30]. The probability distribution of the Young’s modulus allows for an estimation of the volume fraction of each phase of C-S-H in the microstructure. In this study, a detailed statistical analysis to obtain values corresponding to the Young’s modulus or volume fraction of various phases is not pursued and only the general trend is discussed.

It is seen from Figure 9 that there seems to be a small increase in the volume fraction of high density C-S-H and a decrease in the volume fraction of low density C-S-H in the mixture with 0.8% CNC compared to the control mixture. Thus, it is realized that the addition of CNC has the potential to modify the nanostructure of hydration product of the mixture as shown from the statistical nanoindentation results. In addition to the nanostructure modification of C-S-H, it is possible that CNC addition could contribute to increased stiffness via the composite effect resulting from CNC’s high elastic modulus [9] and enhanced load-transfer between CNC and the hydration product.

## 4. Conclusions

In this paper, the effect of CNC with a relatively large size on the properties of cement mixtures was examined. The main conclusions are as follows:

- The addition of CNC delays hydration at an early age but then enhances hydration at later ages. The initial delay is most likely due to the adsorption of CNC onto cement particles, reducing the available surface area for the hydration reaction. At later ages, the adsorption of CNC improves the hydration potentially due to the steric stabilization. Although CNC with a larger size than that used in [37] was studied here, the overall trend in the hydration evolution of cement mixtures appeared to be similar;
- The FTIR results indicated the possibility of bond formation between CNC and the hydration product. The results from XRD and FTIR did not indicate the formation of any new phases in the hydration product of the mixture with addition of CNC;
- A slight improvement in the electrical resistivity of the mixtures with CNC compared to the control mixture was observed;
- The statistical nanoindentation measurements showed a small increase in the volume fraction of high density C-S-H and a decrease in the volume fraction of low-density C-S-H in the mixture with CNC compared to the control mixture.

## Figures and Tables

**Table 1:** Chemical compositions of the cement.

Composition	(%)	Composition	(%)
SiO_2_	20.8	Na_2_O	0.2
Al_2_O_3_	5	K_2_O	0.4
Fe_2_O_3_	3.7	SO_3_	2.8
CaO	64.2	TiO_2_	0.2
MgO	0.9	Loss on ignition (%)	2.14
